# Socioeconomic status and self-rated health of Japanese people, based on age, cohort, and period

**DOI:** 10.1186/s12963-016-0095-z

**Published:** 2016-08-02

**Authors:** Hidehiro Sugisawa, Ken Harada, Yoko Sugihara, Shizuko Yanagisawa, Masaya Shinmei

**Affiliations:** 1 Graduate School of Gerontology, J. F. Oberlin University, 3758 Tokiwa-machi, Machida-shi, Tokyo, 194-0294 Japan; 2 Jissen Women’s University, 1-1-49 Higashi, Shibuya-ku, Tokyo, 150-8538 Japan; 3 Tokyo Metropolitan University, 1-1 Minami-Osawa, Hachioji-shi, Tokyo, 192-0397 Japan; 4 Tokushima University, 3-18-15 Kuramoto-cho, Tokushima-shi, Tokushima 770-8503 Japan; 5 Tokyo Metropolitan Institute of Gerontology, 35-2 Sakae-cho, Itabashi-ku, Tokyo, 173-0015 Japan

**Keywords:** Income differences, Health inequalities, Adjusted self-rated health, Age-period-cohort, Model for cross-classified random effects, Japan

## Abstract

**Background:**

Differences in health resulting from differences in socioeconomic status (SES) have been identified around the world. Age, period, and cohort (A-P-C) differences in health are vital factors which are associated with disparities in SES. However, few studies have examined these differences simultaneously. Moreover, although self-rated health (SRH) has been frequently used as an indicator of health, biases in reporting SRH that depend on the socioeconomic characteristics of respondents have been scarcely adjusted in the previous studies. To overcome these limitations, we investigated the associations between disparities in SES and adjusted SRH based on A-P-C, by using a repeated, cross-sectional survey of a nationally representative sample of Japanese people. In addition, we further investigated how exogenous (macroeconomic) conditions unique to a period or cohort would explain trends across successive periods and cohorts.

**Methods:**

Data were obtained from a sample of 653,132 Japanese people that responded to the Comprehensive Survey of Living Conditions (CSLC), which is a cross-sectional survey that had been conducted every three years from 1986 to 2013, on over 10 occasions. In the CSLC, SES has been assessed by household income. We simultaneously controlled for each A-P-C dimension by using the model for cross-classification of random effects, and adjusting SRH data for reporting biases caused by differences in income and A-P-C.

**Results:**

Differences in adjusted SRH associated with income differences decreased with age and reversed after 76 years of age. Period differences indicated that income differences peaked in 1992 and 2007. Moreover, differences in adjusted SRH associated with income differences decreased in periods with high unemployment across all periods. Furthermore, there were no cohort differences in adjusted SRH that were associated with income differences.

**Conclusion:**

In Japan, there are age and period variations associated with adjusted differences in SRH as assessed by income. Moreover, exogenous conditions in each period could help explain periodic trends across successive periods.

## Background

Socioeconomic status (SES) has been identified as a strong social determinant of health in general populations of both developed and developing countries [1–3]. In Japan, studies on health differences resulting from socioeconomic status have occasionally been published since the 1990s [4]. A review article on health differences and risk factors based on SES in Japan indicates significant associations between socioeconomic differences and mortality, morbidity, as well as health-related behaviors, although these differences are less than in the US and Europe [5]. Moreover, differences in SES related to health resulting from Age-Period-Cohort (A-P-C) have been investigated mainly in the US and Europe, whereas the studies conducted in Japan have only investigated associations between age or period with differences in SES related to health.

Certain studies in the US and Europe have shown a decrease in health differences related to SES in later life, whereas other studies have demonstrated a constant increase in health differences related to SES with aging [6]. Findings in Japan on age differences in SES related to health have also been inconsistent [7–9]. Moreover, studies on period differences have accumulated in the US and Europe, and a number of studies have observed widening gaps in health in the past several decades [10, 11]. Also in Japan, some studies have focused on period differences in SES-related health gaps. However, it is uncertain whether SES-related differences in the health of Japanese people have increased, remained stable, or decreased over different periods. Certain studies conducted before 2000 in Japan have shown a decrease in SES-related health differences over time [7, 12, 13], whereas studies conducted after 2000 have indicated different SES-related health trends [14–18]. There are relatively few studies on cohort differences in SES-related health gaps in Japan, although differences by cohorts on SES-related health gaps have been reported in the US and China [19–22].

Studies on differences in SES-related health gaps in health by Age-Period-Cohort (A-P-C) have several limitations. First, only a few studies have simultaneously examined the differences by all three A-P-C variables [9, 15]. The partial usage of the three dimensions could result in biased estimations [23]. Second, although some studies have examined period differences on SES gaps in health, only a few studies have attempted to statistically explain period changes in SES differences in health on the basis of macroeconomic trends, whereas most studies have only discussed the possibility of such relationships [14, 15]. Third, although the majority of studies have used self-rated health (SRH) as an indicator for examining SES-related health differences, few studies have adjusted for reporting biases caused by heterogeneity, or different thresholds across populations with differing demographic or socioeconomic status when they evaluate their own health. According to a review by Fujii et al. [24], threshold levels in SRH depend on sex, age, education, time points, and income. As a result, reporting biases might have caused A-P-C differences in SES gaps in health reported in some studies.

To overcome the limitation of the current method of study, the present study was designed to examine SES differences in SRH, which was adjusted across SES and A-P-C for reporting biases by using a nationally representative sample of Japanese people. The main focus of the study was to estimate independent differences by age, a wider range of periods, and birth cohorts, while simultaneously controlling for each A-P-C dimension. In addition, if large variations in periods or cohorts were observed, we further examined the extent to which exogenous conditions at each time period, or cohort, helped to explain the trends in that period, or cohort, across successive cohorts and periods.

## Methods

### Data

We used data from the Comprehensive Survey of Living Conditions (CSLC), which is conducted by the Ministry of Health, Labour and Welfare of the Japanese government. The CSLC is a nationwide, repeated, cross-sectional survey of households and household members. This large-scale survey started in 1986, and has been conducted every three years since using the same methodology. Complete households and household members living in 5,530 randomly selected area units were sampled in the most recent CSLC survey conducted in 2013, in which trained investigators visited households to distribute and collect self-administrated questionnaires. The total number of households that participated in the survey including the basic information survey was 295,369 (response rate 79.6 %). Survey items included a household questionnaire that inquired about gender, age, having a spouse, and work status, as well as a health questionnaire that inquired about subjective symptoms, difficulties in daily life, and work status. In this study, 36,419 households were selected at random from the complete sample and information was collected regarding their income and savings (response rate 74.4 %). We used micro-data files from each three-year survey conducted between 1986 and 2013. We believe that according to the “Ethical Guidelines for Epidemiological Research” published by the Japanese Government, this study does not need the ethical approval from the ethical review board, because we did not use personally identifiable information (http://www.niph.go.jp/wadai/ekigakurinri/ethical-gl/guidelines.htm). The CSLC is a survey conducted by the Ministry of Health, Labour, and Welfare of Japan for collecting basic statistical data on citizens. Moreover, we obtained permission from the Ministry of Internal Affairs and Communication when requesting access to CSLC data, in order to fulfill confidentiality obligations required by laws in Japan governing the use of official statistical data.

### Study variables

#### Dependent variables

It is has been suggested that SRH can accurately predict future health outcomes, including mortality [25]. Therefore, we used SRH as an indicator of health. We assessed SRH by inquiring, “What is your current health status? Is it excellent, very good, good, fair, or poor?” As described in the section below on statistical analyses, we developed dichotomous variables for responding about SRH after adjusting for differences in SRH threshold levels resulting from income and A-P-C (adjusted SRH), by assigning a scale ranging between 0 (*excellent, very good, or good*) and 1 (*fair or poor*).

#### Independent variables

The main independent variables in this study were four distinct dimensions that included SES, age, period, and cohort. SES was assessed by using the square root scale of annual income by dividing household income by the square root of household size. Three major SES indicators; educational attainment, occupational status, and income have been used in previous studies. We used income as the SES indicator, because the CSLC before 2010 did not inquire about educational attainment. It was also considered difficult to evaluate SES by using occupational status in young adults, middle-aged women, and elderly people, because a minority of these groups was engaged in paid work. We divided the respondents into three groups based on their income levels, such that the lowest income level included people within the first income quartile, the middle income level included people within the second and third income quartiles, and the highest income level (which was the reference category) included people in the fourth income quartile. The highest age of participants was 95 years. It was considered difficult to accurately estimate age patterns related to health differences after this age, because only a small effective sample size was available. The lowest age that was coded was 20 years, because only people over 20 years of age responded to questions on SRH in the 1986 CSLC survey. The period was determined as the survey years from 1986 to 2013. Each cohort was determined based approximately on five-year birth cohorts between 1895 and 1990. These cohorts were defined by their mid-point, such that the 1942 cohort for example included individuals born between 1940 and 1944. The last cohort included six years. Identical to age, the earliest cohort was 1895, because it was considered too difficult to estimate accurate cohort patterns related to health differences before 1895 due to the small effective sample size. Individual variables including gender (female-reference), marital status (married-reference, unmarried, divorced/bereaved), participation in the labor force (participant-reference, non-participant), and region of residence (eight regions) were used as covariates.

If a variation in SES-related health differences were observed in a given period, we further examined the relationships between these variations and exogenous conditions in each period. Previous studies have explained patterns of happiness and SRH over time periods through macroeconomic factors, such as per capita Gross Domestic Product (GDP) and the unemployment rate [26, 27]. Therefore, in this study, exogenous factors possibly affecting health patterns in different periods were examined through changes in real per capita GDP and the unemployment rate. Per capita GDP across periods was measured by using one-year changes in the percentage of per capita GDP compared to the previous year, based on data from the Annual Reports of National Accounts [28]. The unemployment rate for each period was obtained from the Labour Force Survey of the Statistics Bureau of the Ministry of Internal Affairs and Communications [29]. We have not described the assessment of exogenous conditions related to cohort variations, such as relative cohort sizes, economic conditions at birth, or infant mortality at birth, because none of the cohorts showed variations in health differences related to SES.

### Statistical analysis

In adjusting for reporting biases due to income and A-P-C when responding to SRH categories, we used the empirical method described by Fujii et al. [24] and Jürges [30], which consisted of constructing a normalized health index ranging from 0 to 1, in which 0 represented the worst health condition and 1 represented the best health condition across income and A-P-C. The health index was based on objective information on health problems, such as physical and mental health problems that had been diagnosed by a physician. The absence of any health condition indicated the best health status, with the presence of a condition reducing the health index by a given amount, or a percentage.

In the case of respondents who had visited doctors or practitioners of acupuncture and moxibustion, CSLC included self-report questions assessing 10 physical and mental health conditions that were diagnosed, including hypertension, stroke, angina and cardiac infarction, among others. Although the responses to these items were self-reported, the responses consisted of relatively more objective information on the health condition than in SRH. Items inquiring about physical and mental health were composed of slightly different categories across CSLC. Therefore, items related to certain physical and mental health conditions were combined to develop identical items across conditions by including 27 categories, such as “other diseases,” “unknown diseases,” “no visit to doctors,” and “no answer.”

We calculated weights of each physical and mental condition (the estimated coefficients) on SRH by estimating a generalized ordered probit regression of SRH on diagnosed physical and mental conditions (reference:”no visit to a doctor”). The weights constrained to be identical for each SRH category. All estimated coefficients in each condition were statistically significant at the 0.1 %. Since the variable on which we based our computation of weights was the SRH itself, the generalized ordered probit ensured that threshold parameters were dependent on income and A-P-C as covariates, such that health reporting thresholds could vary by income and A-P-C. In this study, age was treated as a continuous variable whereas the cohort and period were treated as dummy variables.

We calculated the health index as a linear prediction from the generalized ordered probit regression. The health index for each respondent was calculated by subtracting the value predicted only by the income and A-P-C from the value predicted by all variables including physical and mental conditions, income, and A-P-C. The health index was normalized such that 0 represented the worst and 1 represented the best health condition across income and A-P-C. We computed the SRH category that a respondent would report, given the respondent’s health index, if the respondent were to behave similar to an average CSLC respondent. Each respondent whose health index was in the bottom 1.5 % (the first average threshold) was assigned to poor health, if between 1.5 % and 13.5 % (between the first and second average thresholds) the respondent was assigned to fair health, and so on. We have mainly presented the results of adjusted SRH. However, we also analyzed non-adjusted SRH values and have described differences in the results between the two indicators to examine the necessity to adjust SRH in studies which used SRH as an outcome variable.

A model identification problem could occur when analyzing the differences by age, period, and cohort on aggregate population data by using conventional linear regression models, because of the complete linear dependency among the three variables. Yang and Land [27] has proposed a hierarchical A-P-C (HAPC) modeling approach using individual-level data and a multilevel modeling framework for addressing these types of problems. Access to individual-level observations facilitates the identification of fixed-effects models by creating different time intervals among age, period, and/or cohort, and the addition of quadratic age differences into the equations. Moreover, as period differences occur from temporal, or sequential changes in life conditions and circumstances that basically have an equal impact on all cohorts, and as cohort differences arise from differences in life conditions and experiences over the life course of cohorts, it is possible to conclude that each person in the same time period, or in the same cohort are embedded in the same social historical context. As a result, in HAPC modeling, respondents are simultaneously influenced by two higher-level social historical contexts defined by the time period and cohort, which can model them as random-effects [27]. We specifically adopted a cross-classified, random-effects, two-level HAPC model (CCREM) that could estimate fixed effects of age and its quadratic term as Level-1 factors, and estimate random effects of period and birth cohort by treating these variables as Level-2 factors. In addition, if significant variations were to be observed in period or cohort, we could examine whether changes in exogenous conditions could explain the period and cohort patterns in income differences through the addition of Level-2 covariates to the CCREM framework [27]. Analyses were conducted using GLIMMIX, which is included in the SAS software package.

CSLC samples were selected by multi-stage cluster sampling. If intra-cluster correlations were taken into account, standard errors of coefficient would be underestimated when cluster analysis is conducted. Therefore, generalized estimating equations (GEE) and multilevel models are used as statistical methods to account for intra-cluster correlations. However, because of the large number of clusters, it was considered difficult to enter cohort and period variables as Level-2 clusters. Moreover, multilevel analyses cannot be conducted by using GEE. If the intra-cluster correlation in this data were to have only a small effect on the standard errors of coefficient, it would not be necessary to consider the intra-cluster correlation. In this study, we compared between the results that accounted for the intra-cluster correlation by GEE and those that ignored the intra-cluster correlation by ordinal logistic regression by using the Level-1 equation. This indicated that both results were nearly identical and therefore, we used the mixed-effects regression models without taking the intra-cluster correlation into account.

The slope of the regression line with age might cause an ambiguous mixture of age and cohort, because the centered scores obtained by centering at the grand mean contain both within- and between-cohort variations [31]. However, even when the grand mean is centered, we can obtain an unbiased regression slope in the regression line for age by entering cohort means as a predictor in the Level-2 intercept equation [32].

### Summary statistics

Table 1 shows summary statistics for adjusted SRH, household income, age, period, cohort, and control variables based on the CSLC between 1986 and 2013. The number of respondents over 20 years, or less than 94 years of age that gave information regarding income and savings in the household survey, and 1895–1990 birth cohorts was 709,768. Respondents with at least one missing value in a variable were excluded from the study (*N =* 56,636). As a result, the final effective sample was 653,132 respondents. Respondents that were excluded from the analyses were more likely to be female, have higher SRH, be unemployed, be single, be older, or have a lower income.

**Table 1:** Summary statistics for self-rated health from the Comprehensive Survey of Living Conditions, 1986 to 2013

Variables	Description	n	Unit	
Outcome				
Adjusted	1 = fair or poor,	87,926	%	13.5
self-rated health	0 = excellent, very good, or good	565,206		86.5
Level-1 variables				
Household equivalence income scale	Reference = Fourth quartile 1 = First quartile 1 = Second/Third quartile	163,327 163,279 326,526	%	25.0 25.0 50.0
Age^a^	Respondent’s age at survey year	653,132	Mean (range) SD	49.59 (20–94) 17.08
Sex	1 = man 0 = women	309,409 343,723	%	47.4 52.6
Labor force participation	1 = participation, 0 = non-participation	414,025 239,107	%	63.4 36.6
Marital status	Reference = married 1 = un-married 1 = divorce/bereaved	469,371 108,446 75,315	%	71.9 16.6 11.5
Region of residence	Reference = Kanto 1 = Hokkaido 1 = Tohoku 1 = Chubu 1 = Kinki 1 = Chugoku 1 = Shikoku 1 = Kyushu	158,476 25,901 67,730 131,068 102,158 50,401 27,258 90,140	%	24.3 4.0 10.4 20.1 15.6 7.7 4.2 13.8
Level-2 variables				
Cohort^b^	1895–1899 1900–1904 1905–1909 1910–1914 1915–1919 1920–1924 1925–1929 1930–1934 1935–1939 1940–1944 1945–1949 1950–1954 1955–1959 1960–1964 1965–1969 1970–1974 1975–1979 1980–1984 1985–1990	653 2,399 6,057 12,258 18,268 29,049 43,232 51,299 55,744 63,737 71,276 66,421 55,208 52,311 46,792 37,816 21,701 12,068 6,843	%	0.1 0.4 0.9 1.9 2.8 4.4 6.6 7.9 8.5 9.8 10.9 10.2 8.5 8.0 7.2 5.8 3.3 1.8 1.0
Period	1986 1989 1992 1995 1998 2001 2004 2007 2010 2013	85,998 87,817 81,950 74,324 66,768 57,591 52,043 46,245 46,860 53,536	%	13.2 13.4 12.5 11.4 10.2 8.8 8.0 7.1 7.2 8.2

^a^Centered by grand mean in analysis

^b^The last cohort includes six years

## Results

Table 2 displays the percentages of respondents that rated their health as fair or poor in each age range, time period, and birth cohort by income levels. The percentages were adjusted for reporting biases in SRH. However, these were not adjusted for age-, period-, cohort-differences, or individual-level control variables. Table 2 also displays ratios of odds for rating health as fair, or poor, by those in the first income quantile compared to those in the fourth income quantile. The data for all respondents aggregated over the 10 health conditions indicated large differences in adjusted SRH related to income levels (odds ratio: 1.87). Income differences by age were larger in middle-aged respondents than in younger or older respondents. Period differences indicated that income differences peaked in 1992 and 2007. Moreover, each birth cohort from 1930 to 1944 showed larger income differences than other cohorts.

**Table 2:** Percentage of people that rated their health as fair or poor according to age, period, and cohort for different income levels, as well as odds ratios of the odds for fair or poor in first quantiles to ones in fourth income quantiles^a^

	First quartile	Second/Third quartile	Fourth quartile	Odds ratio
Age				
20–24	0.6	0.3	0.3	2.01
25–29	0.8	0.8	0.6	1.34
30–34	1.5	1.3	1.3	1.16
35–39	2.7	1.9	1.9	1.43
40–44	4.0	2.6	2.8	1.44
45–49	7.0	4.7	4.5	1.60
50–54	9.4	7.0	6.7	1.44
55–59	13.4	10.5	9.8	1.42
60–64	20.0	17.9	16.9	1.23
65–69	27.3	26.4	25.4	1.10
70–74	40.7	41.1	38.8	1.08
75–79	51.9	53.4	52.6	0.97
80–84	55.6	58.5	57.4	0.93
85–89	58.0	57.7	59.5	0.94
90–94	56.8	59.2	59.1	0.91
Period				
1986	11.5	7.7	7.7	1.56
1989	13.8	8.6	8.1	1.82
1992	14.7	8.7	7.9	2.01
1995	15.4	9.7	8.9	1.86
1998	19.2	12.6	10.9	1.94
2001	21.5	15.3	13.1	1.82
2004	25.0	17.6	14.6	1.95
2007	22.2	15.5	12.0	2.09
2010	22.8	16.8	13.2	1.94
2013	27.5	21.1	16.8	1.88
Birth cohort				
1895–1899	37.9	33.7	41.8	0.85
1900–1904	43.3	44.0	45.6	0.91
1905–1909	46.7	47.6	48.2	0.94
1910–1914	46.8	47.9	48.7	0.93
1915–1919	42.6	42.2	43.4	0.97
1920–1924	38.9	36.3	35.3	1.17
1925–1929	33.7	32.1	27.3	1.35
1930–1934	29.5	26.1	18.6	1.83
1935–1939	23.9	19.5	13.2	2.07
1940–1944	16.8	12.6	9.6	1.90
1945–1949	10.1	7.3	7.6	1.36
1950–1954	6.3	4.6	5.7	1.11
1955–1959	3.8	2.8	4.3	0.88
1960–1964	2.9	2.2	2.7	1.08
1965–1969	2.7	1.7	1.6	1.71
1970–1974	1.6	1.4	1.1	1.46
1975–1979	1.6	1.2	1.0	1.61
1980–1984	1.2	0.9	0.9	1.34
1985–1990	1.0	0.7	0.6	1.67
Total	18.3	12.4	10.7	1.87

^a^Adjusted self-rated health was used in this table. Percentages were values without adjustment for age differences, period differences, cohort differences, or individual-level control variables

**Fig. 1:**
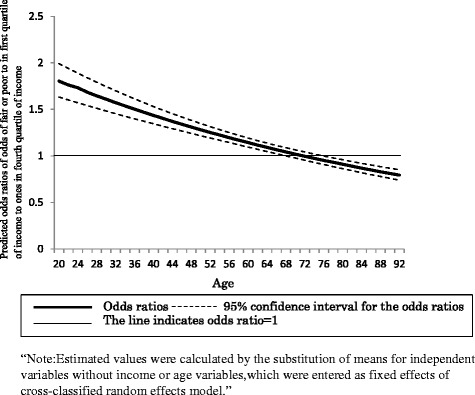
Age differences in income gaps in adjusted self-rated health

Table 3 shows the results of CCREM for adjusted SRH. It can be seen from the table that the interaction between the first quartile of income and age significantly affected adjusted SRH. As shown in Fig. 1, we estimated the odds ratios for odds of rating health as fair, or poor, in the first income quartile relative to the fourth income quartile for ease of interpretation. It can be seen from the figure that the odds ratios decreased with advancing age, as well as the upper limit of the 95 % confidence interval line was below 1 over 76 years of age, suggesting that income differences related to adjusted SRH decreased with advancing age and higher incomes were related to lower adjusted SRH in people at 76 years of age.

**Table 3:** Estimated hierarchical age-period-cohort-models of adjusted self-rated health, 1986 to 2013

Fixed effects	Coefficient	*P* value
Intercept	−2.533	<0.001
First quartile of income (ref = fourth quartile of income)	0.253	<0.001
Second/Third quartile of income (ref = fourth quartile of income)	0.036	0.044
Age (grand mean centered)	0.094	<0.001
Age^2^(grand mean centered)	−0.0004	<0.001
First quartile of income*age (grand mean centered)	−0.011	<0.001
Second or third quartile of income*age (grand mean centered)	−0.002	0.030
Male(ref = female)	−0.105	<0.001
Labor force participation (ref = non-participation)	−0.410	<0.001
Non-marriage (ref = marriage)	0.327	<0.001
Divorce/Bereave (ref = marriage)	0.046	<0.001
Hokkaido (ref = Kanto)	0.172	<0.001
Tohoku (ref = Kanto)	0.057	<0.001
Chubu (ref = Kanto)	0.015	0.252
Kinki (ref = Kanto)	0.146	<0.001
Shikoku (ref = Kanto)	0.109	<0.001
Chugoku (ref = Kanto)	0.154	<0.001
Kyushu (ref = Kanto)	0.018	0.212
Mean of age by each cohort	−0.0001	0.977
Random effects	Variance	*P* value
Period		
Intercept	0.061	0.024
First quartile of income	0.002	0.066
Second/Third quartile of income^a^	-	-
Cohort		
Intercept	0.066	0.003
First quartile of income^a^	-	-
Second/Third quartile of income	0.0001	0.389
Model fit		
-2 Res Log-Pseudo-Likelihood	3883618 (df = 652979.9)	

^a^The results by Cross-Classified Random Effect Model indicated that G-matrix was not positive. As a result, we excluded these variables for the random effect model

**Fig. 2:**
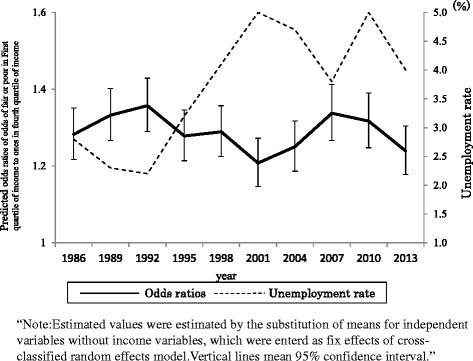
Period differences in income gaps in adjusted self-rated health

Table 3 shows the results of estimated random coefficients model. Level-1 coefficient for the first income quartile showed a marginally random effect across periods (*P =* 0.066). Figure 2 shows estimated random period effects in terms of the predicted odds ratio for odds of rating health as fair, or poor, in the first income quartile, relative to the fourth income quartile across periods. As can be seen in periodic patterns from 1986 to 2013, income differences had a tendency to increase until 1992. Then income differences decreased until 2001 and again increased until 2007. Is it possible to explain these periodic patterns in income differences through macro-socioeconomic trends across periods? Fig. 2 also shows the plots for the unemployment rate across periods. It can be seen from the figure that the plot for unemployment rate is negatively related to periodic trends in income differences in adjusted SRH, such that increases in the unemployment rate reduced income differences while the disparity trend after 2010 seem to be different. We added the unemployment rate to the models as a Level-2 covariate to examine whether the unemployment rate could explain periodic variations in income differences affecting adjusted SRH. The results are shown in Table 4. It can be seen from Table 4 that periods with a high unemployment rates affected the less steep slopes of the first income quartile, suggesting that such periods led to a decrease in income-related differences in adjusted SRH. Moreover, period-level covariates accounted for reduced periodic variances in the first quartile of income (45.5 %). Periods with yearly changes in the percentage of per capita GNP did not have a large impact on slopes of the first quantile of income (omission of results).

**Table 4:** Cross-Classified Random Effects Model of adjusted self-rated health: period level covariates^a^

Random effects		
Period	Variance (*p* value)	Variance (*p* value)
Intercept	0.061 (0.024)	0.062 (0.024)
First quartile of income	0.002 (0.066)	0.001(0.134) % Reduction ^b^ =45.5
Period level covariates		Coefficient (*p* value)
Unemployment rate		−0.031 (0.027)
Model fit		
-2 Res Log-Pseudo-Likelihood	3883618 (df = 652979.9)	3883560 (df = 652932.7)

^a^Cross-Classified Random Effects Model was conducted using all variables in the model shown in Table 3. However, this table shows only related results

^b^Compared to variance estimates from the model without covariates

Table 3 shows that Level-1 coefficients of the first income quartile did not have a significant random effect across cohorts. When we analyzed the data by using unadjusted SRH, we obtained nearly the same results for age and period differences in health differences related to SES. However, Level 1 coefficients of the first income quartile had a marginally significant random effect across cohorts (*P =* 0.064). These results were different from those for adjusted SRH.

## Discussion

In this study, we used aggregate data from over 10 repeated cross-sectional surveys and demonstrated that income levels influenced adjusted SRH. Results showed that odds ratios for odds for fair or poor health in first the quantile to those in the fourth income quantile was 1.87. This finding is quite similar to the results of previous research. The results of the current study also suggest that income differences in health decreased with age. In Western countries, whether or not SES differences in health in the general population increase with age is being debated. Also in Japan, it has been unclear whether SES-related health differences increase or decrease with age. The current study overcame the limitations of previous studies by adjusting reporting bias of SES, using aggregated data, and controlling for confounding factors, such as the cohort and the period. Therefore, it is suggested that the results of this study are more valid than those of previous studies. Based on these results, it is concluded that income differences in SRH decreased with age among Japanese people, which, supported the age-as-leveler theory of health differences related to SES. According to this theory, the decreased association between SES and health in later life can be explained by selective mortality and biological frailty [32]. Moreover, a crossover in which people in the fourth income quintiles had higher than the predicted probability of fair or poor health was observed over 76 years of age. A similar crossover in health disparity between less advantaged and more advantaged groups has been observed in several countries, although the age of the crossover was slightly different between the countries [22, 33]. In Japan for example, Liang et al. indicated that more education is associated with higher mortality among Japanese people aged 80 years or older, compared to those between 60 and 69 years of age [34]. Therefore, it is possible that a crossover of income differences in SRH is observed approximately at 80 years of age in Japan and in other countries. Crossover of SES differences in health might be explained by selective mortality [33]. People in less advantaged groups could be affected by disease and disability at an earlier age than those in more privileged groups. The development of disease and disability is expected to result in earlier deaths, leaving survivors with more physical and mental vitality among the less privileged populations than in more privileged populations. As a result, survivors beyond a certain age would manifest a reversal in SES-related health differences.

The results of this study also indicated that there are large periodic variations in the associations between income differences and SRH. In Japan, studies that have examined time trends in SES-related health differences have indicated that health differences could increase, decrease, or remain stable. Nevertheless, different studies have focused on different time periods, age ranges, and health indicators; therefore, identifying generalized tendencies from the results is difficult. Both Hiyoshi et al. [15] and our study employed the same CSLC, and the results of the two studies are somewhat similar, suggesting that the results of this study confirmed the validity of the results by Hiyoshi et al., although the study by Hiyoshi et al. did not adjust for SRH reporting bias.

Few empirical studies have directly examined the link between characteristics of different periods and the health status of individuals in a given period. This study investigated whether periodic differences in adjusted SRH could be explained by macroeconomic variables, such as the unemployment rate, and change in real per capita GDP over the long term. Results indicated that period-level covariates in the unemployment rate accounted for a reduction in periodic variances in the first income quartile (45.5 %), suggesting that periods with high unemployment were associated with reduced differences in adjusted SRH related to income. The traditional perspective on the health impact of economic fluctuations holds that recessions have the negative relationships to health, especially for lower SES groups [35]. However, previous studies have not provided consistent evidence as to whether economic crises reduce or increase the associations between SES and health. There is evidence from Japan, the US, and South Korea that the economic crisis might have increased differences in SES health [15, 33, 36]. On the other hand, studies in Japan by Kondo et al. [14] and Wada et al. [16] and a study in Finland by Valkonen et al. [37] suggest that the economic crisis mainly affected higher SES groups, rather than lower SES groups, and that higher SES groups suffered more negative health influences. Negative economic conditions might have affected the health of Japanese people with a higher SES more than lower SES groups, because of more severe changes in work environment and employment system for higher occupational status workers [16]. In addition, it also could be because social safety nets in Japan provide mechanisms of security to low SES groups. However, this study did not clarify whether the unemployment rate is related to the disparities of trends after 2010. To verify associations between the disparities of trends and unemployment rate from 1986 to 2010, CSLC data conducted after 2013 need to be analyzed.

A large cohort has more people completing their schooling and entering jobs, which leads to negative socioeconomic achievement and psychological well-being [38]. According to Easterlin [38], it is to be expected that competition for jobs, reduced opportunities for promotion and a tight labor market would be more strongly emphasized in large rather than small cohorts, giving the impression that these factors are more detrimental to lower income groups when large cohorts are used in a study. On the other hand, Honjo et al. [9] pointed out that the popularization with a higher education over the last few decades in Japan might have contributed to the increase in health disparities related to SES across cohorts. To date, only a few empirical studies have examined the relationships between cohort characteristics and SES differences in the health in the cohort. One exception is Beck et al. [21], which suggested that the economic conditions of a cohort at the time of birth might explain racial disparities in health in the cohort, whereas RCS did not explain such disparities. The current study conducted in Japan did not show significant cohort differences in income gaps in adjusted SRH, which did not support Easterlin’s theory discussed above [38]. However, when we used non-adjusted SRH, we obtained results that supported Easterlin’s theory (omission of results). Adjusted SRH data are more valid, and has been suggested that using adjusted data in future studies would result in more accurate results.

Several limitations of the current study constrain the interpretations of these findings. First, this study used only cross-sectional data. Selective mortality and admission of older respondents in lower income groups to care facilities for the elderly might play a role in explaining the reduction of income differences in SRH associated with age. In fact, people in such facilities were excluded from the CSLC. It has been suggested that future investigations should use a longitudinal panel survey to obtain data on mortality and admission to care facilities for the elderly, and to test the influences of selective mortality on SRH differences related to income by age. Panel data could provide conclusive evidence on intra individual changes in age and health. Second, we used an income-based indicator to measure SES, and ignored other socioeconomic indicators, such as education, because the CSLC before 2010 did not inquire about education. Each SES indicator is known to have a different relationship to health and therefore, to identify the relationship between SES and health differences, indicators other than income should be utilized for assessing SES. Third, we used the data of 10 surveys conducted every three years to examine the period differences on SES gaps in health. It has been suggested that data collected over longer periods would be needed to obtain a more accurate representation of the differences by periods.

Despite these limitations, this study overcame shortcomings of prior studies that have investigated the differences by period on SES gaps in health, including studies that have been conducted in Japan that had only focused on periodic trends in SES gaps in health and ignored cohort differences. In contrast, this study examined not only period and cohort differences, but also age differences. Furthermore, this study examined A-P-C differences on income gaps in health by simultaneously controlling for A-P-C differences using CCREM. In addition, we examined the extent to which exogenous conditions at each period of time, or cohort, could help explain periodic trends, and trends in each cohort across successive cohorts, or periods. Also, we statistically examined A-P-C difference on SES gaps in health using adjusted SRH data, which increased the validity of our findings.

## Conclusions

Previous studies conducted around the world, including Japan have identified differences in health that are associated with SES. This study provided new evidence of health disparities related to differences in SES. Variations in health differences related to SES were identified according to A-P-C, as well as exogenous conditions in each time period and cohort that could help explain the trends of that period, or the cohort, across successive cohorts, and periods. We used SRH which was adjusted for reporting biases due to income and A-P-C. These trends indicate that differences in adjusted SRH associated with income differences decrease with age. Period differences of income differences between 1986 and 2013 indicated that income differences decreased from 1992 to 2001 and increased until 2007. Moreover, differences in adjusted SRH associated with income differences decreased in periods with higher unemployment across all periods. The cohort differences of income differences related to adjusted SRH were not significant.

## Abbreviations

A-P-C, age, period, and cohort; CCREM, cross-classified, random-effects two-level HAPC model; CSLC, comprehensive Survey of the Living conditions; GDP, gross domestic product; GEE, generalized estimating equations; GNP, gross national product; HAPC, hierarchical A-P-C; RCS, relative cohort sizes; SES, socioeconomic status
